# Supplementary material for: Dual-Labelled Nanoparticles Inform on the Stability of Fluorescent Labels In Vivo
Source: Pharmaceutics. 2023 Feb 25;15(3):769. doi: 10.3390/pharmaceutics15030769 (PMC10059031; doi:10.3390/pharmaceutics15030769)
Supplement: Supplementary file 1 [file pharmaceutics-15-00769-s001.zip › pharmaceutics-2150074-SI.pdf]

Supplementary information

Dual-Labelled Nanoparticles Inform on the Stability of Fluorescent Labels In Vivo

Sabrina Roussel, Philippe Grenier, Valérie Chénard and Nicolas Bertrand *

Faculty of Pharmacy, CHU de Quebec Research Center, Université Laval, 2705 Laurier Blvd, Québec, QC G1V 4G2, Canada

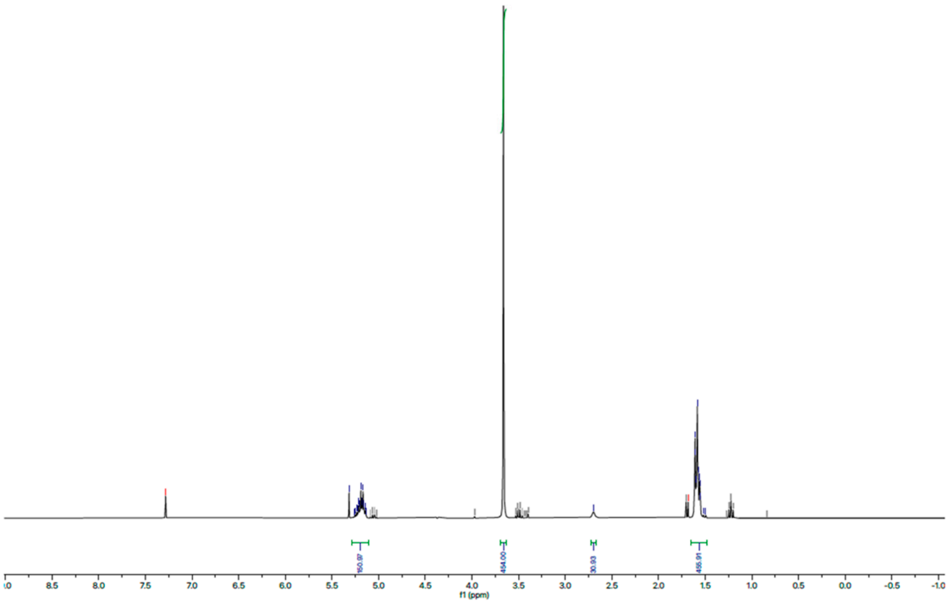

Figure S1. NMR of PEG-PLA copolymer in $CDCl_3$

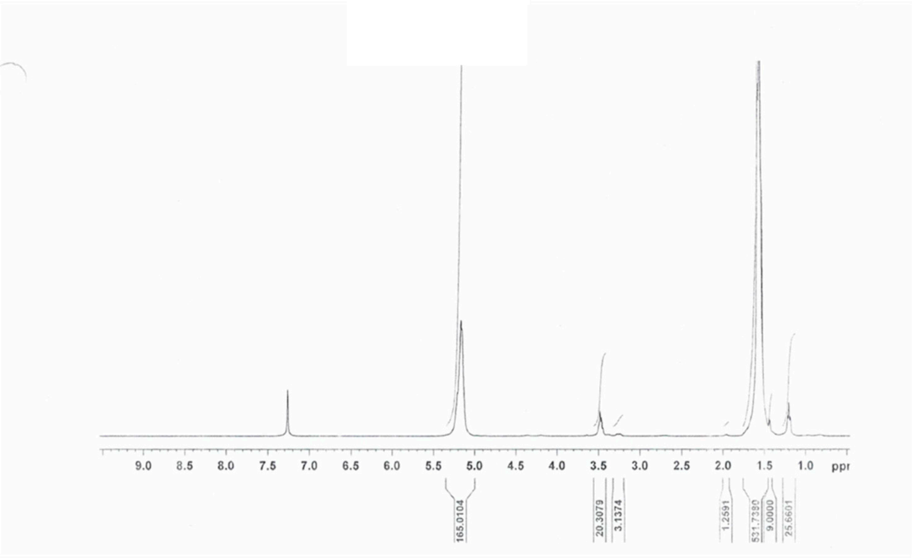

Figure S2. NMR of the tboc-PLA in $CDCl_3$

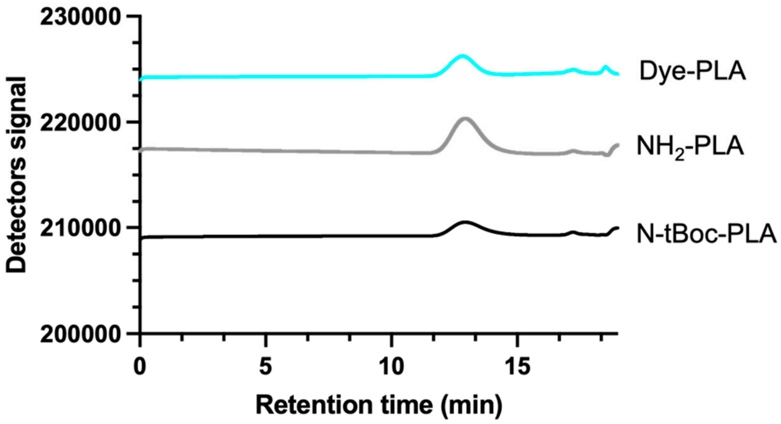

Figure S3. GPC signal of the PLA polymer before removing the protecting group (black) and after deprotection (grey). The blue line represented the PLA-NH_2 once conjugated to a fluorophore showing no difference with the polymer before labelling.

Table S1. Percentage based on the area under the pic related to each phase by HPLC to measure the partition coefficient of free dyes.

Dye	% Octanol	% Water
Cy5	93	7
AZ647	2	98
BODIPY650	99	1

Table S2. Optimization of the nanoparticle's formulation with PEG-PLA, $PLGA_{30k}$ and PLA-dye solution at a concentration of 10 mg/mL

Formulation	PEG-PLA (µL)	$PLGA_{3ok}$(µL)	PLA-Dye (µL)	Speed (RPM)	Water (mL)	Size (nm)	PDI
1	100	0	0	1600	10	41	0.255
2	600	400	0	1600	10	70	0.188

3	400	600	0	1600	10	80	0.176
4	400	600	0	1600	6	100	0.345
5	400	600	70	1600	10	200	0.267
6	400	600	45	1600	8	130	0.359
7	500	400	100	1600	8	180	0.278
8	600	400	100	1600	10	130	0.398
9	600	300	200	1600	10	250	0.456
10	600	0	400	1600	10	150	0.471
11	700	0	300	1600	10	103	0.162

Table S3. Raw data of the partition coefficient between ethyl acetate and water for PLA-dye and free fluorophore

molecules	Water phase	Water phase + 0.1 N NaOH	Water phase + 0.5 N NaOH	Water phase + 1N NaOH	EtOAc	EtOAc + 0.1 N NaOH	EtOAc + 0.5 N NaOH	EtOAc + 1N NaOH
PLA-Cy5	6.43E+04	3.23E+04	1.20E+05	5.50E+04	1.60E+07	1.73E+04	1.73E+04	3.10E+04
PLA-Az647	9.10E+04	5.77E+06	1.33E+07	2.07E+07	2.17E+05	1.04E+06	5.20E+04	1.73E+04
PLA-BDP	1.56E+05	3.70E+04	3.30E+04	6.20E+04	1.43E+07	1.10E+07	1.30E+07	1.17E+07
Cy5	5.23E+06	1.77E+06	2.20E+06	2.93E+06	3.77E+07	2.43E+07	4.60E+07	4.90E+07
Az647	4.80E+07	4.60E+07	2.77E+07	1.73E+07	4.06E+05	3,52E+05	1.90E+05	7.50E+05
BDP	5.47E+04	2.60E+04	1.37E+04	1.77E+04	6.20E+07	1.20E+07	1.90E+07	4.90E+07

Table S4. Density of PEG on the surface for all the different nanoparticles

	Cy5	Az647	BDP
Density PEG/100 nm^2 (IV)	45	52	43
Density PEG/100 nm^2 (IP)	49	-	42

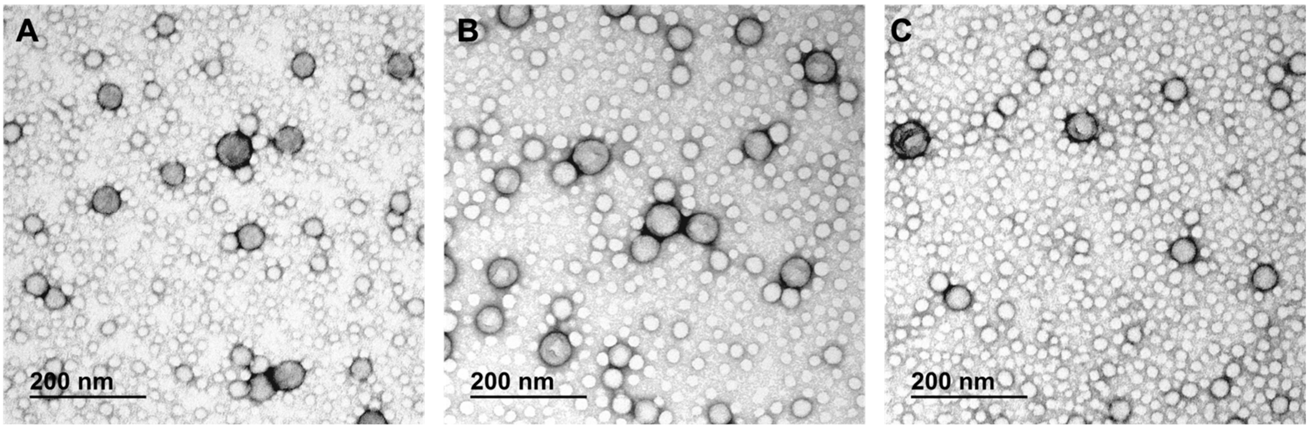

Figure S4. Transmission electronic microscopy of the nanoparticles. A) BD650, B) Cy5, C) AZ647.

Table S5. Biodistribution profile in the organs after i.v injection for radioactivity and fluorescence (15 mg/mL)

Time (h)	% ID Radioactivity			% ID fluorescence		
	Cy5	Az647	BDP	Cy5	Az647	BDP
0.25	93 ± 2	84±8	90±12	92±9	67±27	148±17
0.5	91 ±3	75±11	83±11	80±3	53±20	118±10
1	83±7	70±8	76±8	56±2	44±19	87±4
2	68±17	66±5	69±8	35±6	22±7	75±4
4	70±4	57±7	56±6	32±5	11±2	41±9
6	60 ±5	51±6	54±6	23±7	7±2	34±7
12	43±2	39±1	41±4	18±2	5±2	23±5
24	19±11	18±4	19±3	9±3	-1±3	9
AUC (%ID.h.g $^{-1}$)	1112±88	1016±43	1037±104	531±50	203±39	759±91
Kel (h $^{-1}$)	0.0853± 0.0009	0.092±0.001	0.092±0.005	0.20±0.03	0.4±0.2	0.2±0.1

Table S6. Biodistribution profile in the organs after intraperitoneal injection for radioactivity and fluorescence (15 mg/mL)

Time (h)	% ID radioactivity		% ID fluorescence	
	Cy5	BDP	Cy5	BDP
0.25	10 ±4	4±2	3±1	5±1
0.75	33 ±10	26±5	23±4	29±6
1.5	53±13	36±9	37±11	41±9
3	56±11	50±5	62±3	54±1
6	52±9	34±11	46±8	45±4
12	41±7	29±7	28±7	37±9
24	18±3	18±1	10±4	22±7
AUC (%ID.h.g $^{-1}$)	821±146	620±124	623±74	763±110
Cmax (% ID)	56±12	50±5	62±3	54±1
Tmax (h)	3±1	3	3	3
Biodisponibility (%)	79 ± 10	67± 11	117± 14	101± 12

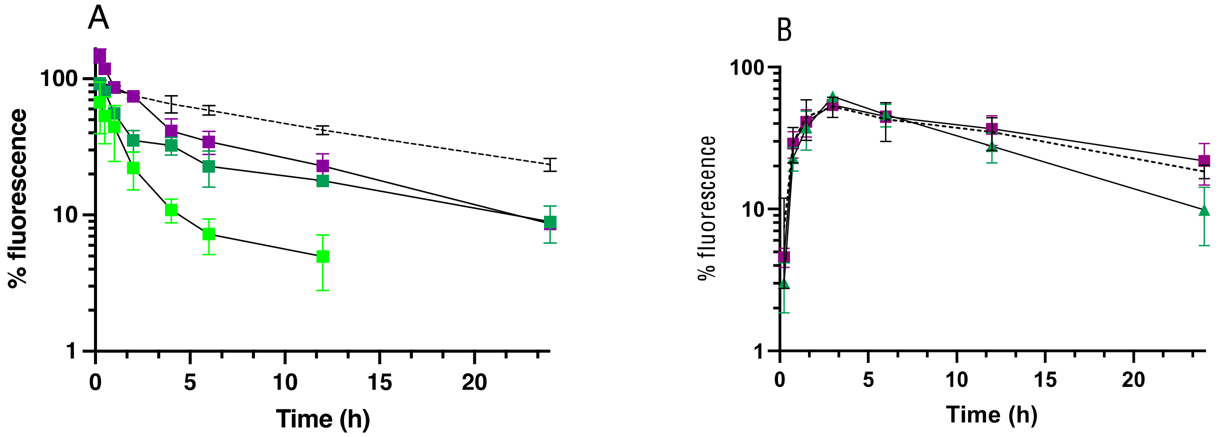

Figure S5. a) Pharmacokinetic of the dual-labelled nanoparticles after IV injection. B) Pharmacokinetic by IP route. Data represented a mean ± SD (*n=3*). Light green represented AZ647, dark green is Cy5 and purple is BODIPY650. The data line represented the average of the signal obtain by radioactivity analysis.

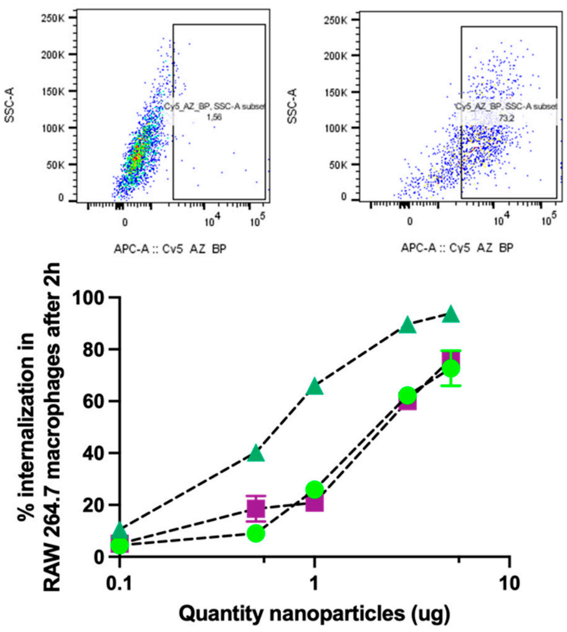

Figure S6. . Macrophage RAW 264.7 internalize differently the nanoparticles based on the dye in a concentration dependent manner. Light green circle represented AZ647, dark green triangle Cy5 and purple square is BD650. Data represent a mean ± SD (*n=3*).

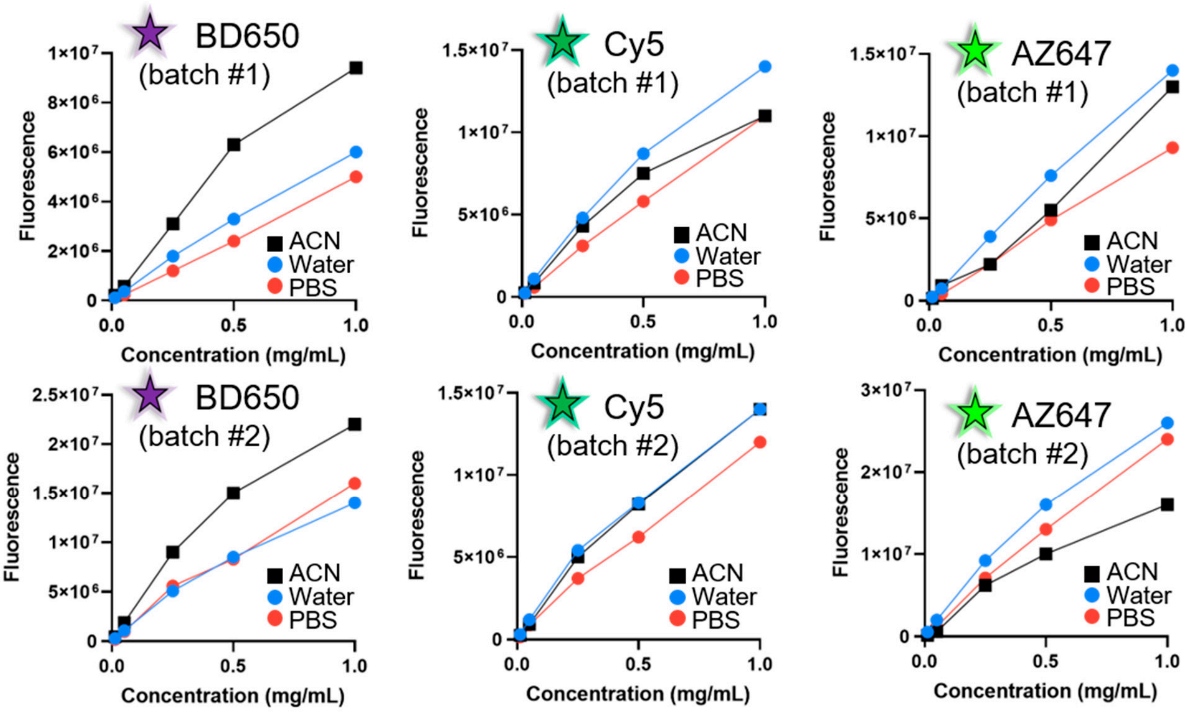

Figure S7. Calibration curve of the nanoparticles in different condition. Blue represented water, red PBS, yellow plasma and black acetonitrile. A is Cy5, B is AZ647 and C is BODIPY650.

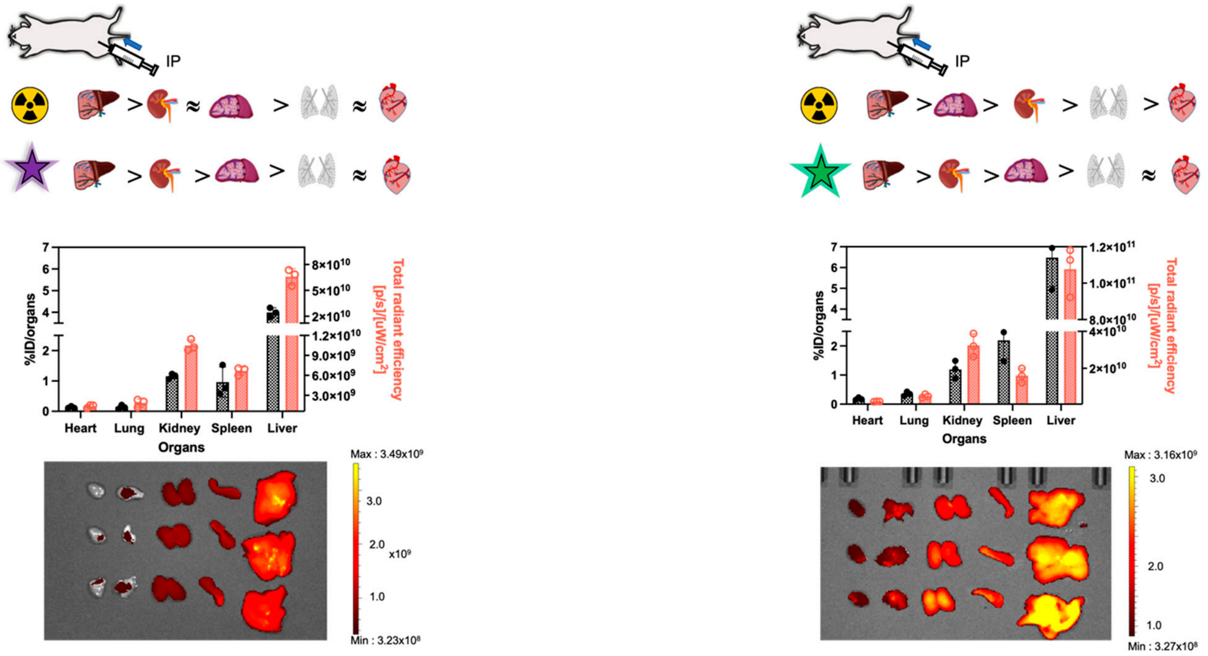

Figures S8. Biodistribution pattern based on the fluorescence (right axis) and radioactivity (left axis) after intraperitoneal administration. Black represented the value relative to the left axis (radioactivity) and red the value based on fluorescence (right axis). Data represented

a mean ± SD ($n = 3$). On the left it's for BD650 and on the right is Cy5. The organs on the IVIS picture are represented as follow (from left to right) : heart, lung, kidney, spleen and liver.

Table S7. Analysis of the urine of all the mice pull together after the last time point of the pharmacokinetic for Cy5 and AZ647

Fluorophore	Fluorescence (radiant efficiency)	Radioactivity (DPM)
Cy5	1.3×10^9	5430
AZ647	2×10^9	5789
